# Deep Learning-Based Automatic Modulation Classification Using Robust CNN Architecture for Cognitive Radio Networks

**DOI:** 10.3390/s23239467

**Published:** 2023-11-28

**Authors:** Ola Fekry Abd-Elaziz, Mahmoud Abdalla, Rania A. Elsayed

**Affiliations:** 1 Electronics and Communications Engineering, Zagazig University, Zagazig 44519, Egypt; mabdallah@zu.edu.eg (M.A.); rania_ahmed@zu.edu.eg (R.A.E.); 2 Department of Electronics and Communications Engineering, October 6 University, 6th of October City 12585, Egypt

**Keywords:** automatic modulation classification, raw IQ sequences, deep learning, convolutional neural network, and wireless channel impairments

## Abstract

Automatic modulation classification (AMC) is an essential technique in intelligent receivers of non-cooperative communication systems such as cognitive radio networks and military applications. This article proposes a robust automatic modulation classification model based on a new architecture of a convolutional neural network (CNN). The basic building convolutional blocks of the proposed model include asymmetric kernels organized in parallel combinations to extract more meaningful and powerful features from the raw I/Q sequences of the received signals. These blocks are connected via skip connection to avoid vanishing gradient problems. The experimental results reveal that the proposed model performs well in classifying nine different modulation schemes simulated with different real wireless channel impairments, including AWGN, Rician multipath fading, and clock offset. The performance of the proposed system systems shows that it outperforms its best rivals from the literature in recognizing the modulation type. The proposed CNN architecture remarkably improves classification accuracy at low SNRs, which is appropriate in realistic scenarios. It achieves 86.1% accuracy at −2 dB SNR. Furthermore, it reaches an accuracy of 96.5% at 0 dB SNR and 99.8% at 10 dB SNR. The proposed architecture has strong feature extraction abilities that can effectively recognize 16QAM and 64QAM signals, the challenging modulation schemes of the same modulation family, with an overall average accuracy of 81.02%.

## 1. Introduction

Automatic modulation classification (AMC) is the core step of non-cooperative wireless communication systems in which receivers have no prior information about received signals. The AMC system plays a fundamental role in many civil and military applications. It is considered the key technique for building an intelligent cognitive radio system for dynamic spectrum access [1]. Conventional AMC approaches are classified into two types: likelihood-based (LB) approaches and feature-based (FB) approaches [2]. LB approaches depend on the likelihood ratio test (LRT), where the modulation classification problem is formulated as a multiple composite hypothesis testing problem based on signal constellation points and parameters, such as signal amplitude, phase, and noise power. The number of hypotheses depends on the number of modulation schemes to classify [3]. The LB modulation classification depends on the calculation of the probability density functions (PDFs) of the received waveform. Maximum likelihood rest [4], average likelihood ratio test (ALRT) [5], generalized likelihood ratio test (GLRT) [4], and hybrid likelihood ratio test (HLRT) [6] are the main methods studied in the literature. Table 1 shows the summary of different LB approaches with comparison criteria probability of correction classification (P_cc_).

These approaches achieve optimal performance if there is prior knowledge of the channel and signal parameters, which is a lake in most applications. However, obtaining these parameters provides higher computation complexity, which is the main drawback of these approaches. The computational complexity of these methods stems from the total number of required operations. In ML (maximum likelihood) detection, without knowing which modulation symbol the signal sample r[n] belongs to, the likelihood is calculated using the average of the likelihood value between the observed signal sample and each modulation symbol Am. The joint likelihood of several observed samples is calculated by the product of all likelihood of individual samples. These values are calculated during the classification process; therefore, the computation complexity should be included as a part of the classifier. In [4], ML modulation detection was used to recognize six different modulation schemes, including BPSK, QPSK, 8PSK, 4QAM, 16QAM, and 64QAM. The total calculations are given by the following:

(1) $$Multiplication=5NI\sum_{i=0}^{I}Mi,$$

(2) $$Addition=6NI\sum_{i=0}^{I}Mi,$$

(3) $$Exponential=NI\sum_{i=0}^{I}Mi,$$

where N number of samples being classified among modulations schemes I. Mi denotes the alphabet size of the ith modulation candidate. Conventional FB approaches have two steps: feature extraction and classification. Various handcraft features have been utilized in FB methods. The most extracted features from the received signal are instantaneous features [7], higher order cumulants (HOCs) [8], cyclostationary features [9], and wavelet features [10]. For the classification step, support vector machine (SVM) [11], decision tree (DT) [12], and K-nearest neighbor (KNN) [13] are considered the most used classical classifiers in traditional FB algorithms. The performance of the conventional FB methods is mainly affected by the extracted handcraft features and the applied classifiers; therefore, these methods have limited performance in a complex wireless communication system. Deep learning (DL), a branch of machine learning, has attracted attention due to its powerful automatic feature extraction. Recently, deep learning has been applied for automatic modulation classification (AMC) because it performs well and achieves high classification accuracy, especially at a high signal-noise ratios (SNR) and when many modulation types are used. DL-based AMC automatically handles feature extraction and classification together for extracting more compelling features. Several deep-learning-based AMC architectures are being designed, including convolutional neural networks (CNNs) and recurrent neural networks (RNNs) [14]. The authors of [15] proposed an AMC model using two layers of 128 long short-term memory (LSTM) cells and one dense layer. The authors of [16] proposed a hybrid model which combines LSTM and residual neural network (ResNet). The authors of [17] suggested AMC based on depth LSTM architecture, where the modulated digital signal is fed directly to the system. Traditional convolutional neural network architectures, such as GoogleNet [18] and ResNet [19], are widely applied for image processing which applies large filter sizes and deep layers. These architectures need modification to be suitable for raw signal data classification. The convolutional neural network has a powerful feature-learning ability, which is considered an efficient method for modulation classification. CNN-based AMC with image representation started in [20]. The authors transformed the received signal into a 227 × 227 binary constellation image using AlexNet to classify four modulation schemes. In [21], they used enhanced gray and three-channel constellation images. Then, the images were fed into two convolutional neural network (CNN)-based DL models, AlexNet and GoogleNet. In [22], the authors proposed network based on the Inception-ResNet network with changing kernel sizes. The authors of [23] proposed an AMC modulation classification based on dense layer dropout CNN (DDrCNN). The modulation schemes are classified by the IQ sequence representation of the received signal. When SNR is 2 dB, the classification accuracy reaches above 97%. In [24], CNN with four pooled convolutional layers and two fully connected layers has been designed for automatic modulation recognition for raw IQ sequences. At an SNR of 18 dB, their model achieved an accuracy of 98.47%. The authors of [25] proposed a SCGNET network for AMC which used two different types of convolutional layers to improve the classification accuracy to 94.4% at 20 dB SNR without increasing network complexity. The authors of [26] designed a bottleneck and asymmetric convolutional model for the classification of different signals. The architecture has ten convolutional layers, three pooling layers, and a fully connected layer. This model achieves an accuracy of 94.97% at 20 dB SNR. In [27], they proposed a convolutional neural network with Stem and Inception modules. The Stem module has four blocks with a convolutional layer, ReLU layer, batch normalization, and a MaxPooling layer. The Inception module is like the Inceptionv3 network [28] with filter size modification. Table 2 contains previous DL-based AMC techniques.

Although existing DL-based modulation classification methods improve the modulation classification accuracy at high SNR values, some of these models do not achieve an accuracy of more than 95%. In [15], the classification accuracy is limited to 90% at high SNRs. The maximum classification accuracy achieved by model in [16] is 92% at 18 dB SNR. Most recent DL-based AMC models provide a level of confusion regarding QAM16 and QAM64 schemes, the challenging modulation schemes of the same modulation family, as the former is a subset of the later, as in [15,26]. Some of the approaches cause the classification accuracy to exceed 95% at high SNR values, but this is at the expense of increasing the computational complexity in terms of number of trainable parameters and the inference time, as in [23,24,27]. It can be seen from the worldwide literature that the recognition rate of radio modulation based on DL achieves a low recognition rate of SNR below 0 dB, which decreases with the decrease in SNR. Generally, the recognition rate for SNR above 10 dB is higher (about 90%). However, the recognition rate of SNR below 0 dB is very low (generally less than 50%). Therefore, further research is needed in order to improve the recognition rate of SNR below 0 dB.

### 1.1. Problem Statement

In non-cooperative communication systems, automatic modulation recognition is an essential technique in intelligent receivers to recognize various modulation types. Deep learning-based modulation classification achieved significant improvements and promising results for monitoring and controlling the radio spectrum.

Recently, DL-based AMC approaches are designed to improve the accuracy at high SNR values, which is not convenient in realistic scenarios. At low SNR values, the signals have various impairments, including AWGN, multipath fading, and Clock Offset, which make modulation classification at low SNRs a challenging target.

### 1.2. Our Contribution

This paper proposes a novel CNN architecture for robust automatic modulation classification for raw IQ sequences of the received signals under various SNR values. The main contributions of our proposed model are summarized as follows:

- Design a novel convolutional neural network architecture based on a proposed convolutional block with a skip connection that remarkably improves classification accuracy at low SNR below 0 dB compared with state-of-the-art models. Thus, the proposed model is convenient in realistic scenarios.
- The proposed architecture has strong feature extraction abilities, which improves the discrimination of 16QAM and 64QAM which are challenging modulation schemes in DL-based AMC models.

The remainder of the paper is structured as follows: Section 2 describes the signal model and the dataset generation used in this work. The proposed model is presented in Section 3. Performance analysis of the proposed system and a comparison of existing systems are presented in Section 4. Section 5 concludes the paper and presents future work avenues.

## 2. Signal Model and Dataset Generation

### 2.1. Signal Model

**Figure 1:**
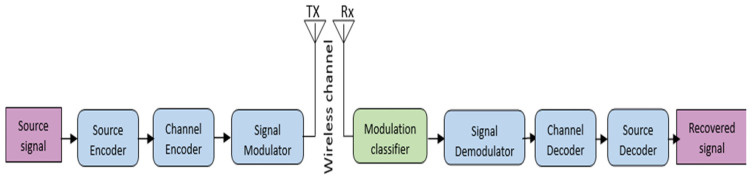
Wireless communication system.

The received modulated signal *S*(*t*) can be formulated using Equation (4):

(4) $$S\left(t\right)=\left(Ai+jAq\right)e^{j\left(2\pi \left(fc+\Delta f\right)\left(t-\varepsilon T\right)+\Delta \theta \right)},$$

where *Aq* and *Ai* are the quadratures and in-phase components (I/Q) of the signal, respectively; Δ*f* is the carrier frequency offset due to oscillator imperfections; Δ*θ* is the phase offset caused by the distance between transmitter and receiver; and *εT* is time due to sampling rate offset, where *T* is the symbol period. Due to channel impairments, signals are affected by several noises among them, of which additive white Gaussian noise (AWGN) and Rician fading are the most common noises. According to these noises, the received wireless signal can be expressed using Equation (5):

(5) $$S\left(t\right)=\left(A_{i}+jA_{q}\right)e^{j\left(2\pi \left(fc+\Delta f\right)\left(t-\varepsilon T\right)+\Delta \theta \right)}\sum_{k=1}^{n}a_{k}\left(t\right)e^{-j2\pi \tau_{k}\left(t\right)}+n\left(t\right),$$

where *n*(*t*) is AWGN; n is the number of paths due to the Rician fading channel; *a_k_*(*t*) is the of gain the k-th path; and *τ_k_*(*t*) is the gain of the *k*-th delay. Figure 1 shows the wireless communication system. The goal of the modulation classifier is to automatically recognize the modulation type from the received signal *S*(*t*), which is an essential step for signal demodulation in non-cooperative communication systems. Amplitude-shift keying (ASK), phase-shift keying (PSK), frequency shift keying (FSK), pulse amplitude modulation (PAM), and quadrature amplitude modulation (QAM) are commonly used modulation schemes [29]. These modulation types are generally generated by modifying one of the carrier signal properties, such as frequency, amplitude, or phase, based on the message signal.

### 2.2. Dataset Generation

For the simulation test, MATLAB 2020b is used to generate the dataset, which includes nine modulation schemes: BPSK, QPSK, 8PSK, 16QAM, 64QAM, GFSK, CPFSK, PAM4, and B-FM. A total of 1000 frames are generated for each modulation class, with a frame length of 1024 I/Q samples. To increase the diversity of the dataset, each frame was impaired with an independent channel. For each frame, the channel applies the following impairments:

- Clock offset which has two effects on the received signal: frequency offset and sampling offset. The former is determined by the clock offset and center frequency (*f_c_*) and the latter by the clock offset and sampling rate (*f_s_*).
- Rician multipath fading is based on path delays, average path gains, Kfactor, and maximum doppler shift.
- Additive white Gaussian noise with an SNR range from −20 to 18 dB and with a 2 dB interval.

**Figure 2:**
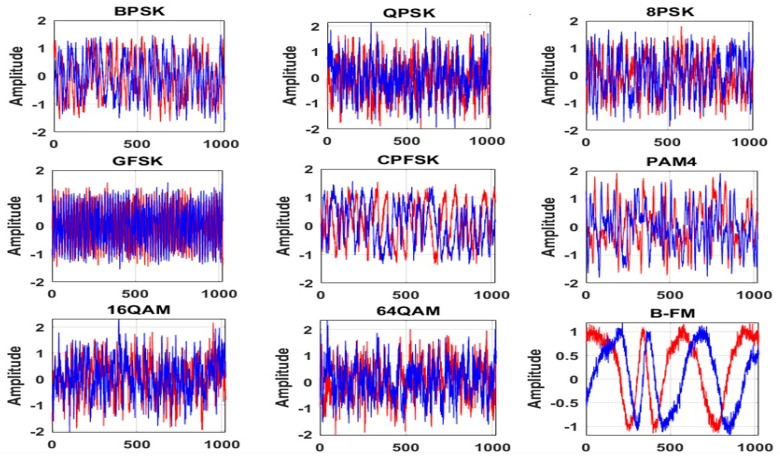
I/Q sequences of the generated dataset at SNR = 18 dB.

As a result, 1000 frames are generated for each modulation type with a size of 2 × 1024 at every SNR value. The modulation parameters of the generated dataset are summarized in Table 3. Figure 2 shows the I/Q sequences of the generated dataset for different modulated signals at an SNR of 18 dB.

## 3. Proposed CNN Model

### 3.1. Fundamentals of CNN Architecture

Like other neural networks, CNN is composed of an input layer, an output layer, and hidden layers in between. The hidden layers are categorized into two types: feature learning layers and classification layers. Feature learning layers include three main layers, convolutional layer, non-linear activation layer, and pooling layer, which are repeated. The convolutional layer is the most significant layer in CNN, which includes several filters (kernels) with specified sizes. This layer extracts features from the input or other output feature maps in deep layers. Equation (6) represents the two-dimensional (2D) convolutional layer output:

(6) $$Z=\sum_{i}W_{i}\times X_{i}+b,$$

where xi is the input map or the output features from the previous layer; Wi is the weights of convolutional kernels; and b is the added scalar bias.

A non-linear activation layer is applied after the learnable layers to decide which neuron will be activated and transferred to the next layer. Rectified linear unit (ReLU) is one of the most common functions that is applied to each element *Z* where any negative value is scaled to zero. The output of the ReLU layer can be expressed using Equation (7):

(7) $$f\left(Z\right)=\left\{\begin{array}{c} 0\;\;\;\;\;if\;\;\;\;Z<0\\ \;Z\;\;\;\;if\;\;\;\;Z\geq 0 \end{array}\right..$$

The iterative operation of the convolutional layer with its activation layer for input feature map X_n_, and the obtained output feature map X_n+1_ can be described via Equation (8):

(8) $$X_{n+1}=f\left(W_{n}\times X_{n}\right)+b_{n}.$$

The main function of the pooling layer is the down sampling of the feature maps with no learnable parameters. Classification layers include a fully connected layer (FC) and its activation layer (SoftMax), which are commonly used in multiclass classification.

### 3.2. Proposed CNN Architecture

**Figure 3:**
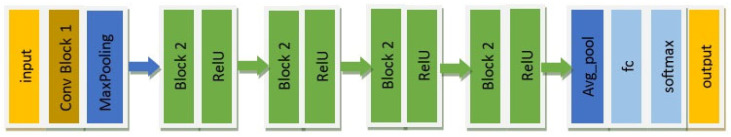
The overall architecture of the proposed network.

**Figure 4:**
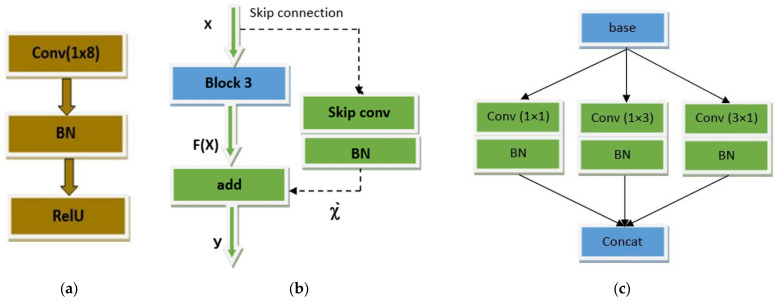
Structures of proposed convolutional blocks: (**a**) Conv block 1; (**b**) block 2; (**c**) block 3.

In this paper, we proposed a novel robust architecture of a convolutional neural network for the AMC of nine modulation schemes at different SNR levels. As shown in Figure 3, the proposed AMC network includes two different convolutional blocks for feature extraction of the signals with different modulation modes. The first block processes the signals with a dimension of 2 × 1024 from the input layer and extracts the feature map of these signals. With a 1 × 2 kernel, the max-pooling layer was then applied, which down-samples the previous feature map by extracting the most prominent features. Subsequently, the extracted features were applied to four blocks of block 2, where each block was followed by its activation layer ReLU. Before the classification step, an average pooling layer is used, which reduces the dimension of the feature map to prevent overfitting. Finally, the fully connected layer and SoftMax layer were added sequentially for classification. Figure 4 shows, in detail, the construction of the basic building blocks of the proposed network.

In Figure 4a, the conv block 1 includes three layers: the convolutional layer with 32 kernels of 1 × 8 size to extract meaningful features from each symbol in the signal; the batch normalization (BN) layer to stabilize and accelerate the training process; and the ReLU activation layer, which is used along with BN layer to prevent vanishing gradient problem.

Figure 4b shows the structure of block 2, which is based on a skip connection via the addition of the actual input of convolutional block 3 and the output of this block. This shortcut connection prevents degradation problems in deep networks such that when *F*(*x*) tends to zero, our model still has non-zero weights and continues to learn. Mathematically, this can be expressed as Equation (9):

(9) $$y=F\left(x\right)+\overset{`}{x}.$$

**Figure 5:**
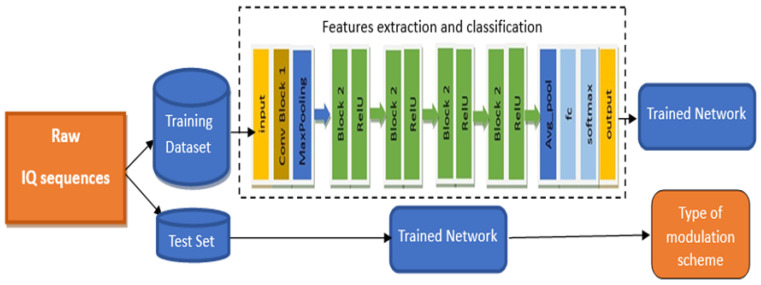
Block diagram of the proposed system.

It also prevents the vanishing gradient problem while updating the weights in backpropagation, resulting in improving the overall accuracy of the proposed model. In the shortcut path, a convolutional layer associated with the batch normalization layer is put to match the input/output dimensions of block 3. The structure of block 3 is shown in Figure 4c, which includes three parallel branches. Each branch has a convolutional layer of 32 kernels with a stride of (1, 2) followed by a batch normalization layer. In the proposed network, three convolutional layers with asymmetric kernels of sizes 1 × 1, 1 × 3, and 3 × 1 are used to extract more meaningful and powerful features. To increase the convergence of the proposed network, these layers are organized in parallel. The feature maps from the three branches are concatenated at the end of each block. Table 4 shows the detailed description and configuration of the proposed network which includes the output size of each layer and the filter size for each convolutional layer. The block diagram of the proposed automatic modulation classification system is shown in Figure 5.

## 4. Experimental Result and Analysis

### 4.1. Network Training

The generated dataset was randomly split as follows: 80% for training, 10% for validation, and 10% for testing. With random initial weight, the proposed network was simulated, trained, and tested from scratch with an Intel Core i7 processor, 16 GB RAM, and a single NVIDIA GeForce RTX 2070 GPU using MATLAB 2020b. The training hyperparameters of the proposed network are listed in Table 5.

### 4.2. Training Complexity

During training, the performance of the model is evaluated on the holdout validation dataset. To avoid overfitting and improve the generalization of the proposed network to be more useful for predictions of new data, we used early stopping of the training based on validation performance such that the training stops when the validation loss has not improved for 10 validations. After early stopping, the model with the best validation loss is loaded for evaluation on the holdout test dataset. The proposed model was trained and tested 10 times; then, the model with the best performance in terms of classification accuracy was selected. The model with the best accuracy was trained for approximately 147 min with a stochastic gradient descent optimizer over a 144,000-frame training set with a batch size of 128. Algorithm 1 below indicates the process of the proposed system.
**Algorithm 1:** Proposed AMC system**Input**: Dataset (raw I/Q sequences)**Results**: modulation type**Procedure:****Step 1:** Randomly divide the dataset into 80% for training, 10% for validation, and 10% for testing;**Step 2**: Construct the network as shown in Figure 1;**Step 3:** Set the training hyperparameters;**Step 4**: Insert the training dataset into the proposed system;**Step 5**: Train the network with the training dataset;**Step 6:** The model training is evaluated by the holdout validation dataset;**Step 7:** The weights are updated by the SGDM optimizer until the validation loss is not improved.  Load the model with the best validation loss.**Step 8:** Train the proposed model 10 times;**Step 9:** Apply the testing frames to each trained network;**Step 10**: Select the trained model with the best classification accuracy;**Step 11:** Recognize the modulation scheme;**Step 12:** Calculate network accuracy.

### 4.3. Classification Accuracy Comparison

The performance of modulation classification is measured via accuracy metric, which can be calculated as follows:

(10) $$Accuracy=\frac{TP+TN}{TP+TN+FB+FN}$$

where TP, TN, FP, and FN are the true positive, true negative, false positive, and false negative, respectively.

**Figure 6:**
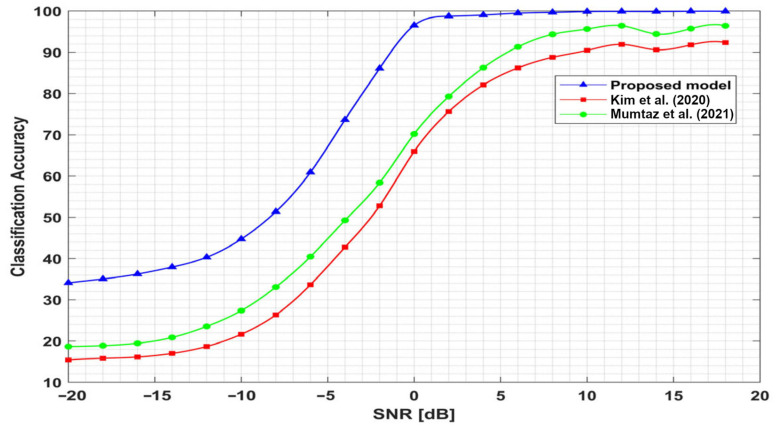
Comparison of overall classification accuracy; [26,27].

To verify the performance of our proposed model, the classification accuracy of the proposed network was compared with two models from the state of the art. The models presented in [26,27] were adopted as comparison algorithms. For a fair comparison, these models were trained with the same training dataset and evaluated on the same holdout test dataset. As shown in Figure 6, the proposed model has outstanding recognition accuracy overall compared to models [26,27]. It has significant improvement in the recognition rate at low levels of SNR. At an SNR of −10 dB, the classification accuracy of the proposed model is higher than the accuracy of the models [26,27] by 23.1% and 17.3%, respectively. In addition, at high signal-to-noise ratio values, the proposed model also outperforms these models. At an SNR of 10 dB, our proposed model achieves an accuracy of 9.4% and 4.2% higher than the accuracy of models [26,27], respectively.

**Figure 7:**
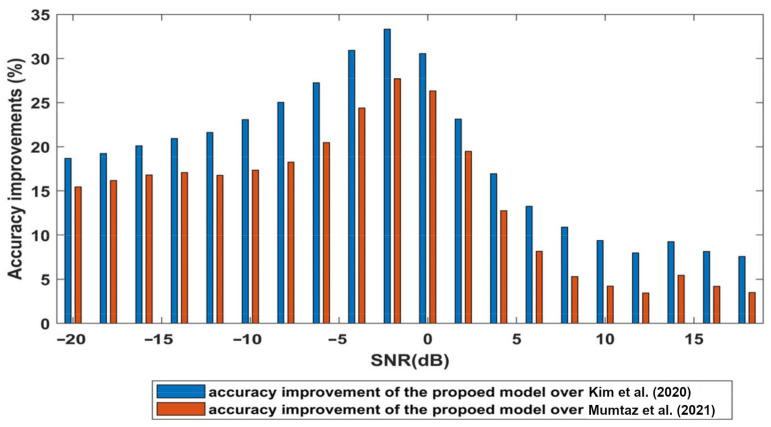
Accuracy improvements of the proposed model; [26,27].

The accuracy improvements of the proposed model over the other two models are shown in Figure 7. The blue bars indicate the improvement in accuracy of the proposed architecture over model [26] at all SNR values; the red bars show the improvements over model [27]. Hence, the proposed model outperforms these models along all SNR values, especially at low levels of SNRs. Thus, our proposed model has excellent robustness in non-cooperative communication systems.

### 4.4. Confusion Matrix Comparison

**Figure 8:**
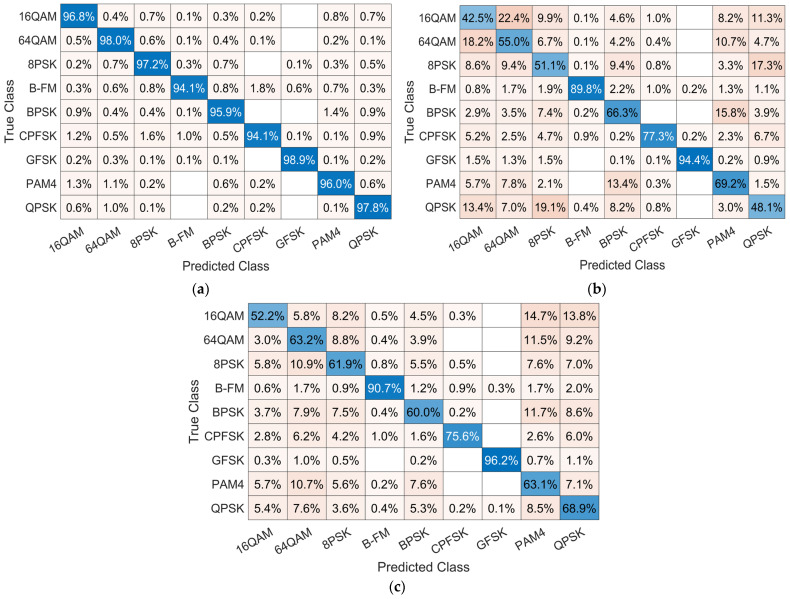
Confusion matrix of the three models at SNR of 0 dB: (**a**) confusion matrix of the proposed model; (**b**) confusion matrix of model [26]; and (**c**) confusion matrix of model [27].

**Figure 9:**
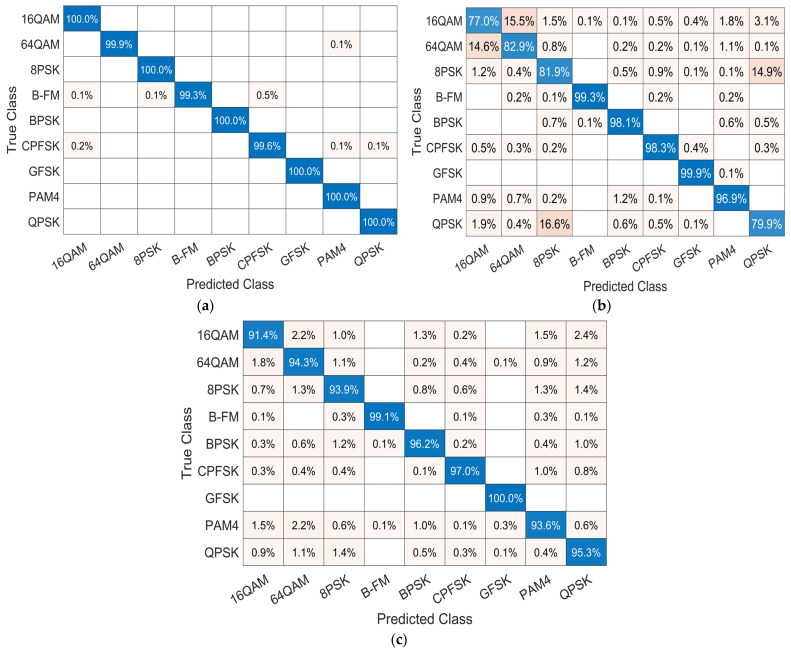
Confusion matrix of the three models at SNR of 10 dB: (**a**) confusion matrix of the proposed model; (**b**) confusion matrix of model [26]; and (**c**) confusion matrix of model [27].

The confusion matrix is an effective evaluation metric that evaluates the effectiveness of the proposed model. The vertical column shows the actual label of the modulated frames, and the horizontal row shows the label predicted by the network. It indicates detailed results of normalized classification accuracy for each modulation type at a certain SNR value. Figure 8 and Figure 9 illustrate the confusion matrix for each model at 0 dB SNR and 10 dB SNR, respectively. Figure 8a shows that the proposed model achieves more than 90% classification accuracy for all modulation types. As shown in Figure 8b, model [26] recorded a recognition accuracy on GFSK and B-FM of about 90% and becomes easily confused between these pairs of modulation schemes: 16QAM and 64QAM; and QPSK and 8PSK. Figure 8c shows that model [27] achieved an accuracy of more than 90% on GFSK and B-FM with a general accuracy for other modulation types. The classification accuracy of the three models improves with the increase in SNR value to 10 dB as shown in Figure 9. However, the model in [26] is still easily confused between 16QAM and 64QAM and between QPSK and 8PSK, as shown in Figure 9b.

### 4.5. Learned Features Visualization

**Figure 10:**
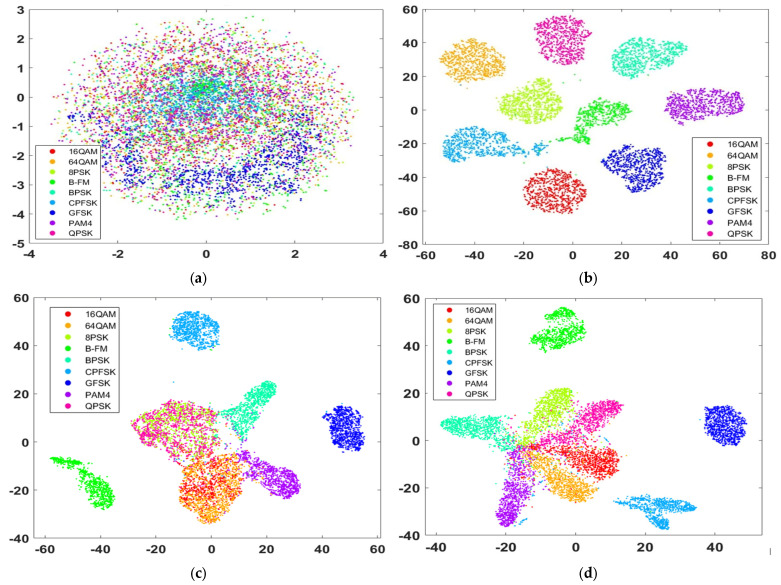
Learned features visualization by t-SNE of the three trained models at 10 dB SNR: (**a**) visualization of input data; (**b**) learned features visualization of the proposed model; (**c**) features visualization of the model [26]; and (**d**) learned features visualization of the model [27].

This section provides a visualization of the output-learned features of the three models to illustrate how these models can differentiate among different modulation schemes. Figure 10 shows the input data visualization and the three models’ learned features at 10 dB SNR. For learned features visualization, we use the t-SNE (t-distributed stochastic neighbor embedding) method [30], a non-linear dimensionality reduction method, which maps the output learned features of the trained model into 2D features for convenient visualization. To show the closest features that resemble each class, the learned features of the pre-Softmax layer (fully connected layer) are visualized. As shown in Figure 10b, the learned features of the proposed model are the most separable in terms of their modulation types. Figure 10c shows the visualization of the learned features of model [26]. There are obvious overlaps between 16QAM and 64QAM (red and orange clusters) and between QPSK and 8PSK (pink and green clusters); however, the learned features of model [27] have much clearer boundaries, as shown in Figure 10d. As we can see, the visualization of the learned features is a good reflection of the classification results for the three models.

### 4.6. Computational Complexity Comparison

Table 6 shows the computation complexity of each model, including the total number of parameters and the inference time per frame. From the table, it is obvious that our proposed model has about 47% fewer trainable parameters than the model [27] in addition to achieving higher classification accuracy with lower prediction time compared to this model. Model [26] is computationally lower than the proposed model with lower prediction time because the layer depth of this model is not deep; however, this is at the expense of classification, which is well illustrated in the previous section.

### 4.7. Individual Classification Accuracy

**Figure 11:**
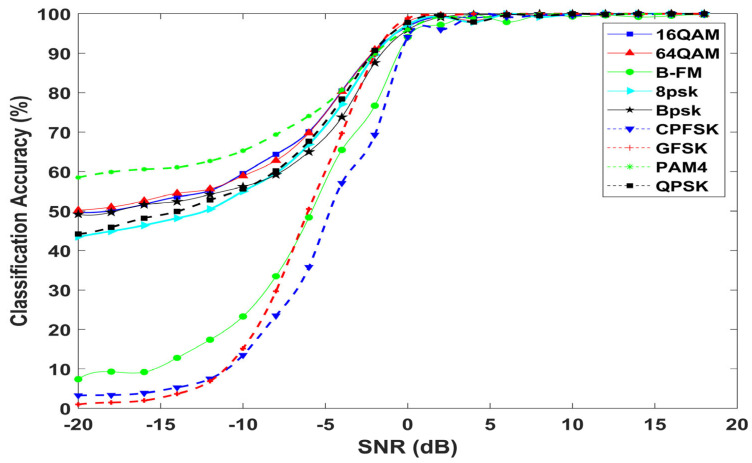
Classification accuracy of individual modulation schemes.

Figure 11 shows the individual classification accuracy of the proposed model for each modulation scheme at different SNRs. The classification accuracy of all modulation types increased with increasing SNR and saturated close to 100% when the strength of the signal is dominant with respect to noise at 0 dB. As shown in Figure 11, the proposed model achieves remarkable classification accuracy at low SNR values for 16QAM, 64QAM, PAM4, BPSK, QPSK, and 8PSK signals. The proposed model recognizes 16QAM and 64QAM signals, with an accuracy of 64.4% and 69.8%, respectively, at −8 dB SNR. For the three modulation types (GFSK, CPFSK, and B-FM), the proposed model provides low classification accuracy at low levels of SNRs, which increases with increasing SNR values.

### 4.8. Effectiveness of the Proposed Architecture

**Figure 12:**
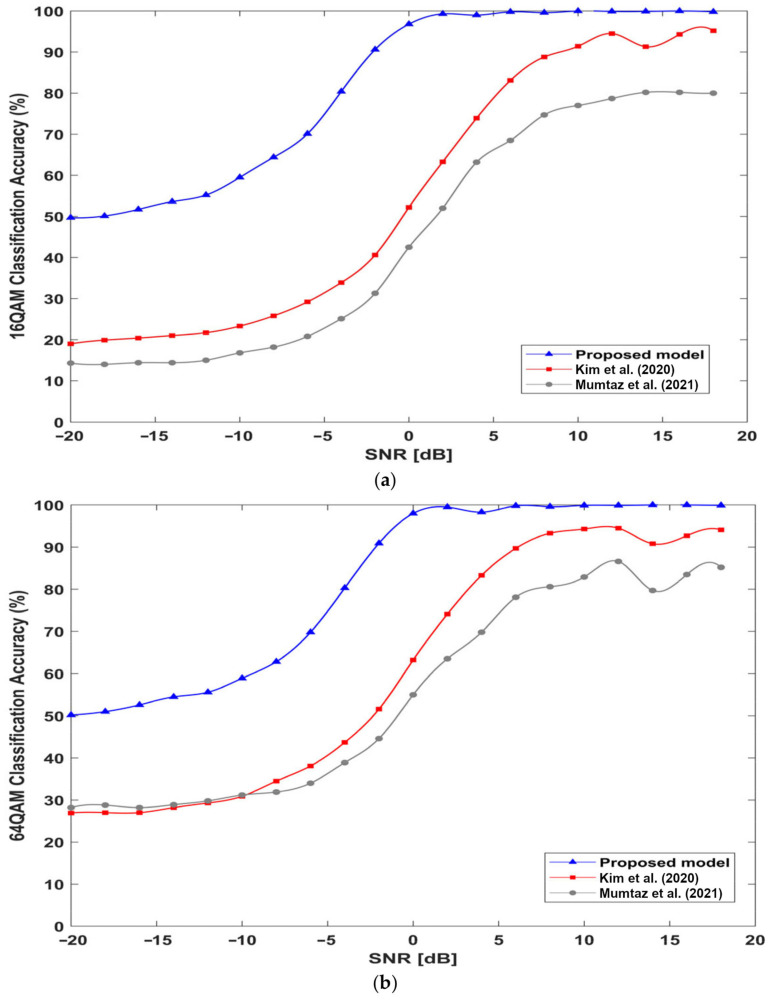
QAMs classification accuracy: (**a**) 16QAMs classification accuracy of the three models; (**b**) 64QAMs classification accuracy of the three models; [26,27].

Figure 12 shows that our proposed model has strong feature extraction abilities that can effectively recognize 16QAM and 64QAM signals, the challenging modulation schemes of the same modulation family, with an average accuracy of 81.02%. Figure 12a shows the classification accuracy for the 16QAM signals of the three models. The proposed model can identify the 16QAMS signal with an accuracy of 49.7%, 70.1%, and 90.6% at −20 dB, −6 dB, and −2 dB, respectively, and the recognition increases to 100% with an increase in SNR, which achieves an overall average accuracy of 80.97%. The models in [26] and in [27] can recognize the 16QAM signals with an overall average accuracy of 44.065% and 54.14%, respectively. The classification accuracy for 64QAMS signals of the three models is shown in Figure 12b. The proposed model achieves the highest overall average accuracy of 81.057%. The model in [26] predicts 64QAM signals with an overall average accuracy of 54.47%. The model in [27] recognizes 64QAMS signals with an overall average accuracy of 60.36%. These results reveal the superiority of the proposed architecture.

### 4.9. The Performance of the Proposed Model Versus the Model Hyperparameters

**Figure 13:**
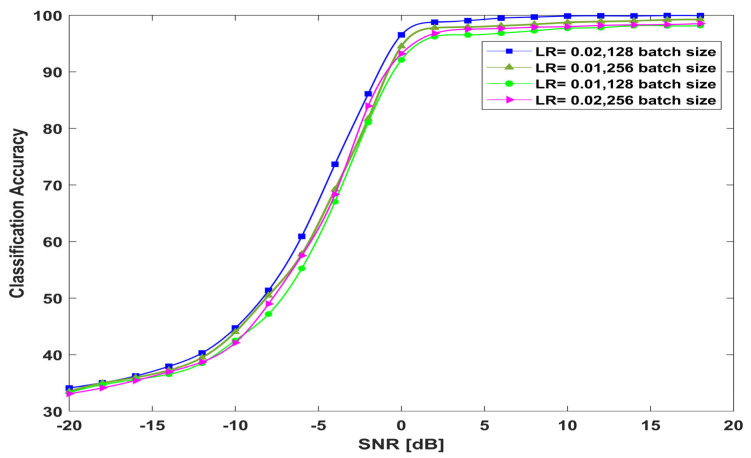
The performance of the proposed model versus hyperparameters.

This section provides the performance of the proposed architecture at different hyperparameters. Figure 13 shows the classification accuracy of the proposed model from −20 dB to 18 dB SNR values with different batch sizes and learning rates.

### 4.10. Ablation Study

The objective of the ablation study is to investigate the effect of changing the model components and hyperparameters on the overall performance to determine the optimal model structure. In this section, we describe each performed modification.

#### 4.10.1. Number of Proposed Convolution Blocks

**Figure 14:**
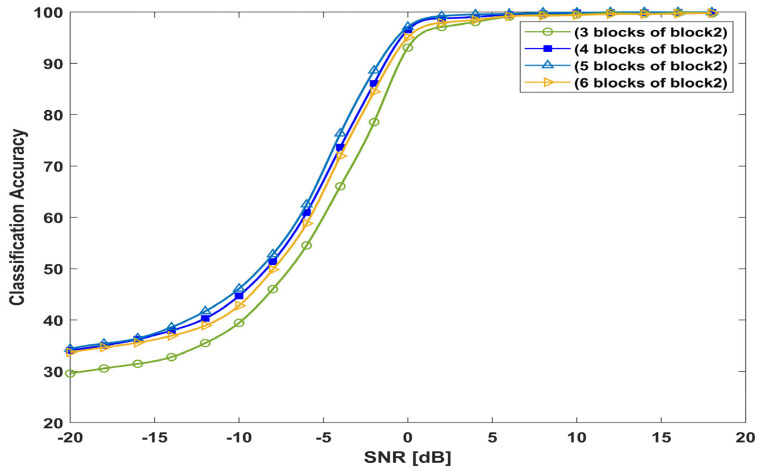
Performance of the proposed model with different numbers of block 2.

To investigate the performance of the proposed convolutional block 2, the classification accuracy of the proposed model is measured using different numbers of blocks. As shown in Figure 14, the proposed model with three blocks of block 2 performs worse in terms of accuracy, especially at low levels of SNRs from −20 to 0 dB, due to deep feature loss at multiscale representative maps. The classification accuracy improves by involving four blocks of block 2. However, increasing the block number to five blocks achieves no significant performance improvements but increases the complexity of the network and hence increases the prediction time. By involving more than five blocks, in addition to increasing the complexity and the prediction time, the model performance starts to decrease because more blocks make the backpropagation difficult. From Figure 14, the proposed model is adopted with four blocks of block 2, which achieves the best performance, along with less complexity without performance degradation.

#### 4.10.2. Number of Filters and Kernel Size

**Figure 15:**
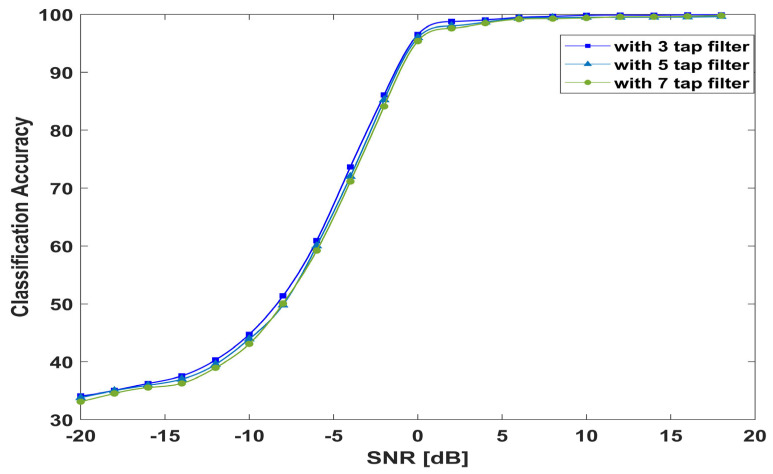
Model performance with different kernel sizes.

To investigate the performance of the proposed model by changing the number of filters and the kernel size. First, the size of the kernels varied from 1 × 3 and 3 × 1 to 1 × 5 and 5 × 1 and then 1 × 7 and 7 × 1. Figure 15 shows that the results are quite similar; the proposed model achieved an average accuracy of 74.65%, 74.06%, and 73.96% with filter kernels of 3, 5, and 7, respectively. Table 7 indicates the number of trainable parameters and the average accuracy at different kernel sizes.

**Figure 16:**
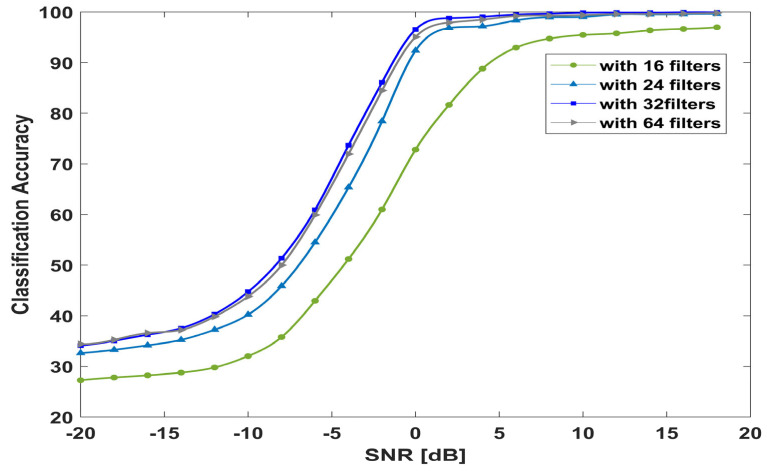
Model performance with different filter sizes.

To investigate the performance of the proposed model with varying filter sizes, we used 16, 24, 32, and 64 filters with kernel sizes of 1 × 3 and 3 × 1. Figure 16 shows that the model with 16 filters in all layers achieves low accuracy over all SNR values. The accuracy starts to increase with 24 filters, especially at high values of SNR. With 32 filters, the model improves the classification accuracy at low and high levels of SNR. By increasing the number of filters to 64 filters, the model achieves quite similar accuracy as the accuracy with 32 filters.

## 5. Conclusions and Future Work

This paper proposes a robust model based on a new architecture of CNN for the automatic modulation classification of nine modulation schemes in the presence of different wireless channel impairments, including AWGN, Rician multipath fading, and clock offset. The results revealed that the classification accuracy of the proposed model compared with the other two methods is the highest, along with varying SNR values from −20 dB to 18 dB. The proposed method improves the classification accuracy at low SNRs where the accuracies are 60.9% and 86.1% at −6 dB and −2 dB, respectively; hence, the proposed model is appropriate in realistic scenarios. In addition to high levels of SNRs, the accuracy could be near 100%. The proposed architecture has strong feature extraction abilities that can effectively recognize 16QAM and 64QAM signals, the challenging modulation schemes of the same modulation family, with an overall average accuracy of 81.02%. Summing up these results confirms the proposed automatic modulation classification model’s excellent robustness, which makes it an effective method in non-cooperative wireless communication systems and many applications such as electronic military warfare. In future work, we plan to design a faster model while maintaining the same accuracy at high SNRs and improving the classification accuracy at very low SNRs.

## Figures and Tables

**Table 1:** Summary of LB approaches.

Ref.	Test	Modulation(s)	SNR Range	P_CC_ % at the Lowest SNR	P_CC_ % at the Highest SNR
[4]	ML	BPSK, QPSK,8PSK, 4QAM,16QAM,64QAM	From 0 to 20 dB	80%	100%
[4]	GLRT	BPSK, QPSK,8PSK, 4QAM,16QAM,64QAM	From 0 to 20 dB	55%	100%
[5]	ALRT	BPSK, QPSK	From −7 to 10 dB	80%	100%
[6]	HLRT	BPSK, QPSK,8PSK,16QAM	From −15 to 15 dB	31%	100%

**Table 2:** Summary of DL-based AMC approaches.

Ref.	Technique	Dataset	Performance Evaluation
[15]	Modulation Classification based on long short-term memory (LSTM)	Modified RadioML2016.10a dataset	Accuracy of 90% at high SNRs
[16]	A hybrid model for AMC based on ResNet and LSTM	RadioML2016.10b dataset	Accuracy of 92% at 18 dB SNR
[22]	Modulation classification based on Inception network and ResNet	RadioML2016.10b dataset	Accuracy of 93.76% at 14 dB SNR
[23]	Dense layer dropout-based CNN architecture for automatic modulation classification	Generated dataset with eight modulation schemes	Accuracy of 97% above 2 dB SNR
[24]	Modulation classification based on convolutional neural network (CNN)	RadioML2016.04c dataset	Accuracy of 98.47% at 18 dB SNR
[25]	Three kinds of modules using grouped and separable convolutional layers	RadioML2018.01A dataset	Accuracy of 94.4% at 20 dB SNR
[26]	A bottleneck and asymmetric convolutional structure	RadioML2018.01A dataset	Accuracy of 94.97% at 20 dB SNR
[27]	CNN model with four stacked convolutional blocks and Inception module	Generated dataset with eleven modulation schemes	Accuracy of 90% at 10 dB SNR

**Table 3:** Modulation parameters of the generated dataset.

Parameter	Value
Center frequency *fc*	902 MHz for digital modulation and 100 MHz for an analog one
Samples per frame	1024
Symbols per frame	128
Sampling rate *fs*	200 kHz
Kfactor	4
Max Doppler shift	4 Hz
Max clock offset	5 ppm
Path delays *τ_k_*	[0, 1.8, 3.4]/*fs*
Average path gains a_k_	[0, −2, −10] dB
Carrier frequency offset	Δ*f*
Phase offset	Δ*θ*
Symbol period	*T*
The received signal	*S*(*t*)
AWGN	*n*(*t*)
In-phase components of received signal	*Ai*
quadrature components of received signal	*Aq*

**Table 4:** Proposed network layout.

Layer	Output Size	Remarks
Input	2 × 1024 × 1	
Conv Block 1	2 × 512 × 32	32 × (1 × 8), stride (1,1) Max-pooling (1,2), stride (1,2)
Block2	2 × 256 × 96	32 × (1 × 1), 32 × (1 × 3), 32 × (3 × 1) 96 × (1 × 1), stride (1,2)
		Feature map concatenation
Addition	2 × 256 × 96	Addition layer
Block 2	2 × 128 × 96	32 × (1 × 1), 32 × (1 × 3), 32 × (3 × 1) 96 × (1 × 1), stride (1,2)
		Feature map concatenation
Addition	2 × 128 × 96	Addition layer
Block 2	2 × 64 × 96	32 × (1 × 1), 32 × (1 × 3), 32 × (3 × 1) 96 × (1 × 1), stride (1,2)
		Feature map concatenation
Addition	2 × 64 × 96	Addition layer
Block 2	2 × 32 × 96	32 × (1 × 1), 32 × (1 × 3), 32 × (3 × 1) 96 × (1 × 1), stride (1,2)
		Feature map concatenation
Pool	1 × 1 × 96	Average pooling (2,32)
FC	1 × 1 × 9	Fully connected layer
Softmax	9	

**Table 5:** Training hyperparameters of the proposed network.

Hyperparameter	Value
Optimizer	SGDM
InitialLearnRate	0.02
MaxEpochs	30
MiniBatchSize	128
LearnRateDropPeriod	9
LearnRateDropFactor	0.1

**Table 6:** Computational complexity comparison.

Model	Total Parameters	Inference Time (ms)
Proposed model.	106 k	0.721
Model [26]	46 k	0.698
Model [27]	200 k	0.786

**Table 7:** Trainable parameters and average accuracy with different kernel sizes.

Kernel Size	Total Parameters	Average Accuracy
1 × 3 and 3 × 1	106 k	74.65%
1 × 5 and 5 × 1	147 k	74.06%
1 × 7 and 7 × 1	188 k	73.96%

## Data Availability

The research data can be available via email requirement.
